# Machine Learning Based Classification of Microsatellite Variation: An Effective Approach for Phylogeographic Characterization of Olive Populations

**DOI:** 10.1371/journal.pone.0143465

**Published:** 2015-11-24

**Authors:** Bahareh Torkzaban, Amir Hossein Kayvanjoo, Arman Ardalan, Soraya Mousavi, Roberto Mariotti, Luciana Baldoni, Esmaeil Ebrahimie, Mansour Ebrahimi, Mehdi Hosseini-Mazinani

**Affiliations:** 1 National Institute of Genetic Engineering & Biotechnology, Tehran, Iran; 2 Department of Biology, School of Basic Science, University of Qom, Qom, Iran; 3 Department of Gene Technology, KTH, Royal Institute of Technology, Science for Life Laboratory, Solna, Sweden; 4 CNR, Institute of Biosciences & Bioresources, Perugia, Italy; 5 Institute of Biotechnology, College of Agriculture, Shiraz University, Shiraz, Iran; 6 Department of Genetics and Evolution, School of Biological Sciences, University of Adelaide, Adelaide, Australia; 7 School of Information Technology and Mathematical Sciences, Division of Information Technology, Engineering and the Environment, University of South Australia, Adelaide, Australia; 8 School of Biological Sciences, Faculty of Science and Engineering, Flinders University, Adelaide, Australia; Mediterranean Agronomic Institute at Chania, GREECE

## Abstract

Finding efficient analytical techniques is overwhelmingly turning into a bottleneck for the effectiveness of large biological data. Machine learning offers a novel and powerful tool to advance classification and modeling solutions in molecular biology. However, these methods have been less frequently used with empirical population genetics data. In this study, we developed a new combined approach of data analysis using microsatellite marker data from our previous studies of olive populations using machine learning algorithms. Herein, 267 olive accessions of various origins including 21 reference cultivars, 132 local ecotypes, and 37 wild olive specimens from the Iranian plateau, together with 77 of the most represented Mediterranean varieties were investigated using a finely selected panel of 11 microsatellite markers. We organized data in two ‘4-targeted’ and ‘16-targeted’ experiments. A strategy of assaying different machine based analyses (i.e. data cleaning, feature selection, and machine learning classification) was devised to identify the most informative loci and the most diagnostic alleles to represent the population and the geography of each olive accession. These analyses revealed microsatellite markers with the highest differentiating capacity and proved efficiency for our method of clustering olive accessions to reflect upon their regions of origin. A distinguished highlight of this study was the discovery of the best combination of markers for better differentiating of populations via machine learning models, which can be exploited to distinguish among other biological populations.

## Introduction

Recent advances in life technologies have led to an exponential growth in size and complexity of biological data. The vast variety of molecular methods developed during the last decade has made it possible to screen diversity at large in organismal populations. As a result, a critical need for new analytical tools seems to have emerged to interpret information, understand processes, and to make all this data meaningful. Machine learning methods such as decision tree and Naive Bayesian learning among others provide revolutionary solutions to pattern recognition, classification, prediction and modeling of the biological information [1–3]. These methods represent frameworks for high throughput analysis of data from molecular diversity markers such as microsatellites [4], and have been successfully exploited for making probabilistic predictions in different capacities of life science research, including genetic studies of plants [5,6]. One of the most significant novelties of machine learning methods is finding the best combination of markers in a ranked structure resulting in high accuracy clustering/prediction [7]. However, the practical usefulness of these methods when applied on empirical datasets derived from population genetics assays seems to have received less notice so far than it may deserve [8].

Supervised learning is a major category of machine learning methods, in which items of a collection are assigned to different classes based on a set of attributes and via a series of devised rules. This is unlike unsupervised learning, where no predefined classes are available and the items are investigated only for possible similarities [1,2,5,9].

Decision trees and the Naive Bayesian classifier are two simple but effective methods of classifications based on supervised learning. Decision trees are graphical models illustrating sequential decision making under uncertainty conditions with the aim of finding best possible decisions. They represent outcomes of different combinations of classification decisions and provide values for each outcome on a probabilistic basis. These algorithms are constructed through analyzing a set of existing data of known classes, called training examples, and can be then used for classifying previously unseen examples [10–14]. The Naive Bayesian classifier is developed on the basis of Bayes’ theorem with the assumption of independence for predictors. Since no iterative parameter estimations are required for the construction of the Naive Bayesian algorithm, the model remains simple and is particularly useful with very large datasets. Despite its simplicity, the Naive Bayesian classifier is notably efficient and often outperforms more sophisticated classification methods [15–19].

The olive (*Olea europaea*) is an important agricultural species being cultivated since ancient times in many regions of the old world [20,21]. Unlike most other fruit species, olive has a very large genetic inheritance represented by over 1,200 cultivars and an abundance of wild trees, as well as a considerable number of ancient cultivated forms waiting to be identified and characterized [22]. Moreover, presence of homonyms (i.e. different genotypes with one denomination) and synonyms (i.e. different denominations for one genotype) associated with high genetic diversity makes olive germplasm very difficult to characterize [23,24]. Different molecular markers have been used in the studies of olive genetic diversity [23,25–28], providing us so far with valuable information on domestication processes and relationships among varieties.

Iran is an olive growing country located outside the traditional Mediterranean range of olive. The distribution of olive species throughout the Iranian Plateau follows different patterns, including colonization of pre-desert areas with very limited water availability and sub-saline lands with extreme temperature variations [24]. Several studies have attempted to address genetic makeup of the olive populations in Iran by means of morphological descriptors and molecular markers [22,29–32], and a high level of genetic variability within the Iranian olive germplasm has been documented. Recently, in a comprehensive investigation of the Iranian olive gene pool using a selection of microsatellite, nuclear and chloroplast markers, Hosseini-Mazinani *et al*. characterized microsatellite profile of over 100 olive genotypes from all around Iran, and compared them with a representative pool of Mediterranean olive cultivars [21]. This study revealed an unexpectedly high level of genetic diversity represented by a few varieties currently under cultivation in small favorable areas and a wide set of local ecotypes as well as individuals of wild olive, *Olea europaea subsp*. *cuspidata*.

The amount of data produced in Hosseini-Mazinani *et al*. provides a reliable platform for evaluating markers in terms of their significance for characterizing different olive varieties [21]. In order to provide a quick and solid approach to determine the most indicative markers with the capacity to distinguish olive populations, we used a set of computational methods including data cleaning, attribute weighting, and supervised machine learning. Our objective was to assess general efficiency of machine learning methods in classifying different olive accessions based on a molecular dataset, i.e. our optimal set of microsatellites. We show that the methodology used in this study is highly reliable in classifying olive accessions of separate geographic origins based on an inferred panel of microsatellite markers.

## Materials and Methods

### The data

In this study with the help of data mining tools we investigated genetics similarities and dissimilarities of Iranian olive populations based on 11 microsatellite markers suggested as a consensus panel for olive genotyping [33]. These loci, including DCA-03, DCA-05, DCA-09, DCA-14, DCA-16, DCA-18, EMO-90, GAPU-71B, GAPU-101, GAPU-103A, and UDO-43 (S1 Table), were screened in 267 different olive accessions originating from laboratory experiments of our previous works [21,22,34]. Data were used to investigate diversity across Iranian olive germplasm sampled at different geographical areas of the Iranian Plateau, and to compare with a representative set of Mediterranean accessions [33]. Considering the origins of our total accessions and based on the results obtained from our previous studies, two different experiments for statistical analyses were designed and carried out.

### Data organizing

**Fig 1:**
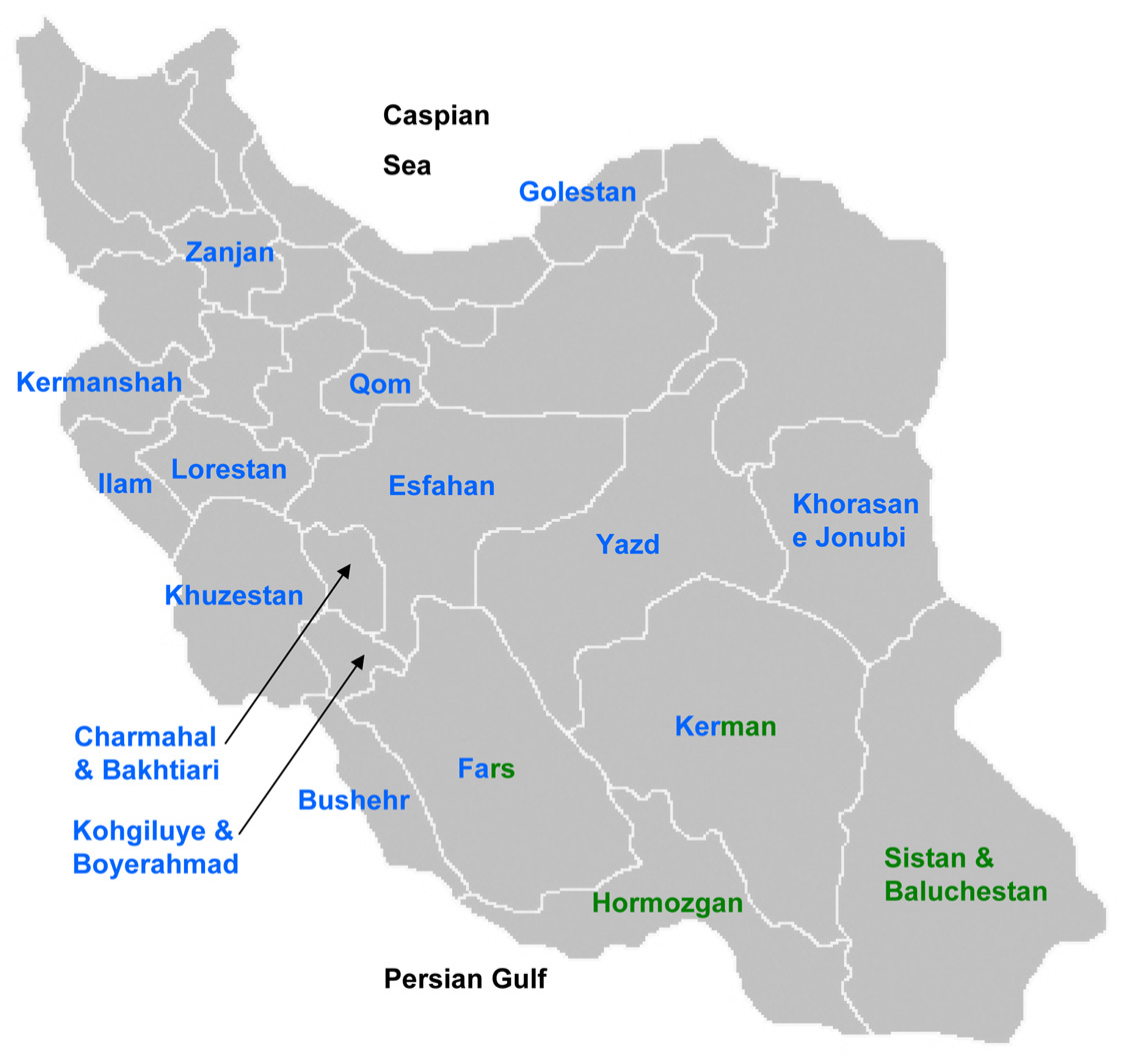
Map of Iran with the main provinces where olive accessions had been sampled. Blue) local ecotypes; green) *cuspidata* specimens.

**Fig 2:**
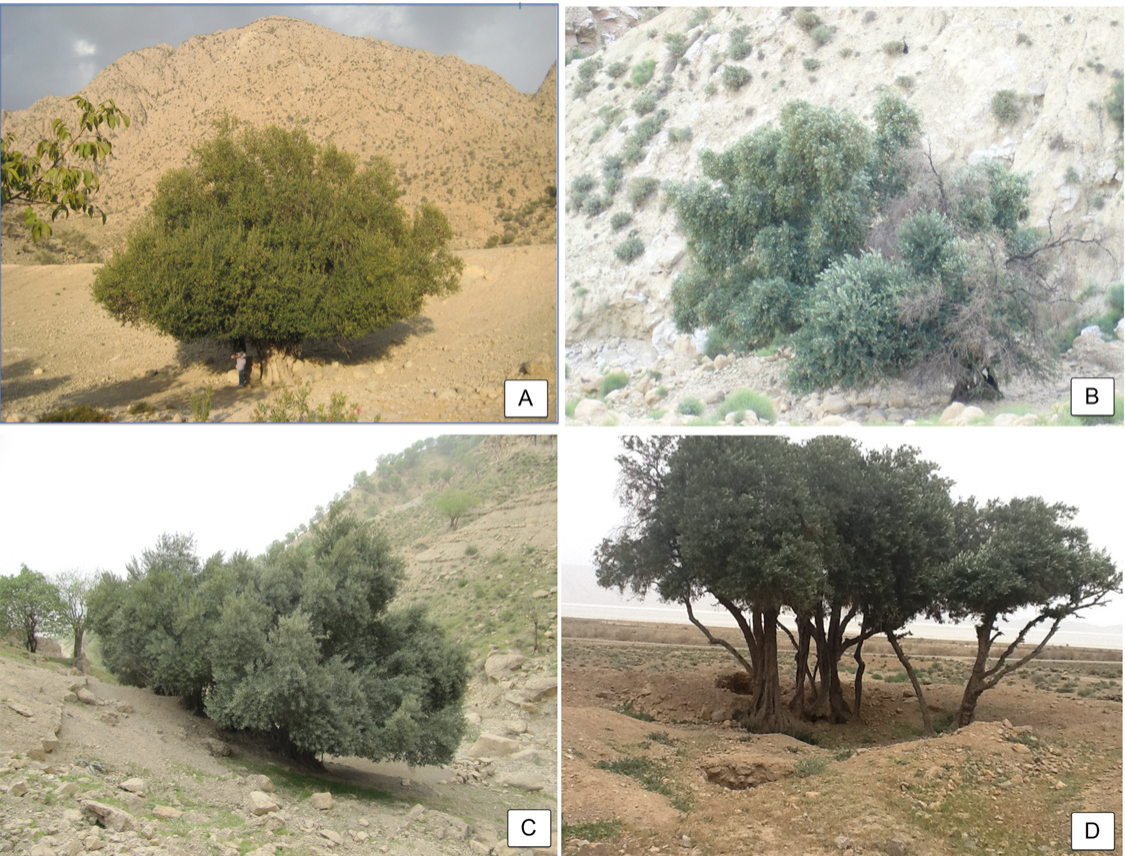
Examples of indigenous Iranian olive. A) Torang *cuspidata* specimen, Kerman; B) Mavi local ecotype, Khuzestan; C) Gardineko local ecotype, Ilam; D) Pirzeytun local ecotype, Fars; adapted from Hosseini-Mazinani *et al*. [36].

In the first experiment, here called the 4-targeted (4-t) experiment, 267 olive accessions were included from four different olive populations as follows: a) 21 reference cultivars grown commercially in different parts of Iran, mostly sampled in a few research stations in the North [21,35]; b) 132 local ecotypes identified from different parts of the country (Figs 1 and 2) [21,22]; c) 37 *O*. *europeae cuspidata* specimens found sporadically at different geographical locations within the southeastern part of the Iranian plateau (Figs 1 and 2) [36]; and, d) 77 Mediterranean varieties from ten Mediterranean countries, selected as the most representative olive cultivars of the Mediterranean basin [33,34]. The first three of these populations including 190 accessions were native to the Iranian Plateau. All 267 olive accessions used in this study had shown different microsatellite fingerprints. We have introduced these four groups of samples as four different targets for machine learning analyses in an experiment called the 4-targeted (4-t) experiment in this study.

In the second experiment, here called the 16-targeted (16-t) experiment, in order to assess differentiating power of the informative loci to distinguish among different olive compartments, only two groups of accessions namely local ecotypes and *cuspidata* specimens were considered for the analysis based on their regions of origin. These 169 accessions originated from 16 of Iranian provinces including Bushehr, Charmahal & Bakhtiari, Esfahan, Fars, Golestan, Hormozgan, Ilam, Kerman, Kermanshah, Kohgiluye & Boyerahmad, Lorestan, Qom, Sistan & Baluchestan, Khorasan e Jonubi, Khuzestan, Yazd, Zanjan (Fig 2). The 16 different provinces abovementioned were also introduced as 16 different targets for machine learning analyses in an experiment called the 16-targeted (16-t) experiment in this study.

### Data analysis

#### Allele identification and allele frequency determination

For each of the 11 microsatellite loci and each of the experiments, total alleles and their frequencies were determined using *GenAlEx 6*.*5* [37]. Allelic profiles for all populations were converted into yes/no binominal variables, assigning ‘yes’ for the present allele and ‘no’ for all other absent alleles at each locus.

The 4-t and 16-t datasets were separately subjected to analytical procedures adopted for data cleaning, feature selection, and machine learning prediction among populations (described below in detail), using *RapidMiner 5*.*3* (Rapid-I, GmbH, Dortmund, Germany).

### Data cleaning

A considerable number of attributes (alleles) were found to be represented privately in certain accessions (particularly within local ecotypes and *cuspidata* specimens populations), and therefore of less significance in characterizing one population versus another. These attributes were removed from the general dataset. Also, some attributes were detected to behave similarly due to being highly correlated. Attributes with strong correlation (Pearson correlation coefficient >0.95) were therefore removed as well to avoid error. The remaining attributes (alleles) created a new dataset, here called the Final Cleaned database (FCdb), which was used as the input source for further selection of alleles (feature selection) through attribute weighting procedure.

### Selection of diagnostic alleles (feature selection)

Feature selection is a common method for identifying significant variables in multidimensional data, typically applied prior to classification and biological interpretation [38]. In order to substantially reduce dimensionality of the data and search for the most indicative predictor attributes (alleles) seven independent attribute weighting algorithms of *Chi Squared*, *Gini Index*, *Information Gain*, *Information Gain Ratio*, *Relief*, *Rule* and *Uncertainty* [39,40] were applied to FCdb. These algorithms gave each attribute (i.e. allele) a weight value between 0.0 and 1.0 depending on its differentiating impact on the target attribute, i.e. the olive populations [41]. The attributes that obtained a weight value equal to or greater than 0.5 by each algorithm were selected and saved as a new Attribute Weighted dataset (AWds). Each newly formed AWds was named after the attribute weighting algorithm that created it. Thus, eight datasets (one new AWds out of each attribute weighting algorithm, plus FCdb) were yielded for each of the 4-t and 16-t experiments, which were all subjected to prediction algorithms, subsequently.

### Machine learning prediction of target populations

In a final step of assessing the classifying methods, we employed two distinct prediction methods of tree induction and Naive Bayes, consisting of 16 and two prediction algorithms, respectively. These models were adopted due to their simplicity, ease of use and clarity of output. All prediction algorithms were independently trained and tested on the eight abovementioned datasets. Accuracy of performance in predicting right group of accessions going together (populations) was evaluated for each algorithm using a 10-fold cross validation procedure. Each of the datasets were shuffled and divided into 10 equally sized sets. The classifying algorithm was trained on 90% of the data, and the remaining 10% was used as an unseen test set to assess efficiency of the classifier. This procedure was repeated 10 times and the average accuracy was calculated. Accuracy was defined by the number of correct predictions over the number of total prediction examples, in percent. Correct prediction meant an example (prediction) for which the value of the predicted attribute was equal to the value of the target (label) attribute. Comparisons of performance among algorithms could also reveal alleles with a key role in assigning populations, besides highlighting the most efficient algorithms and datasets for prediction of unknown accessions for future works.

**Fig 3:**
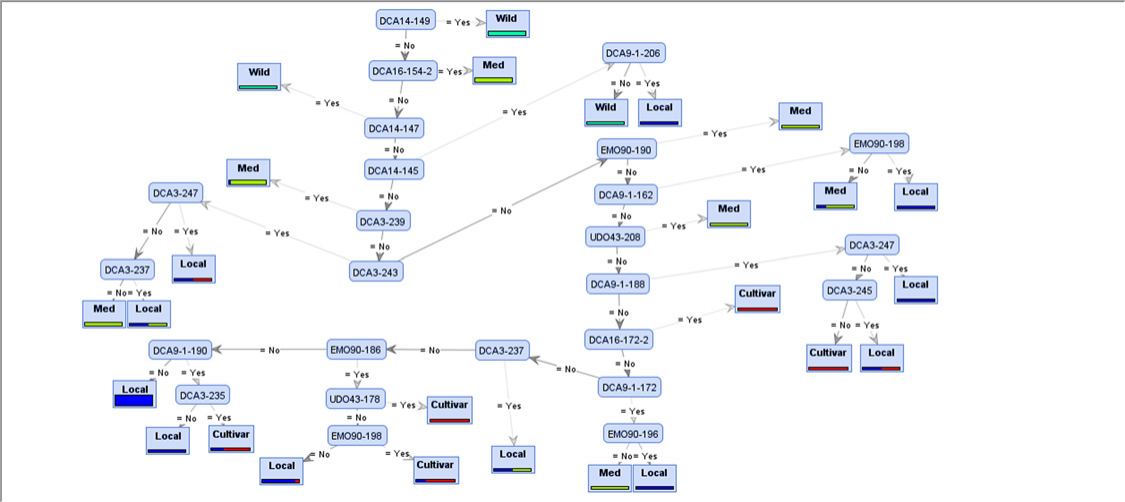
*Decision Tree* generated model showing separation of olive populations in the 4-targeted (4-t) experiment by different alleles. In this model, DCA14-149 was selected as the root.

**Fig 4:**
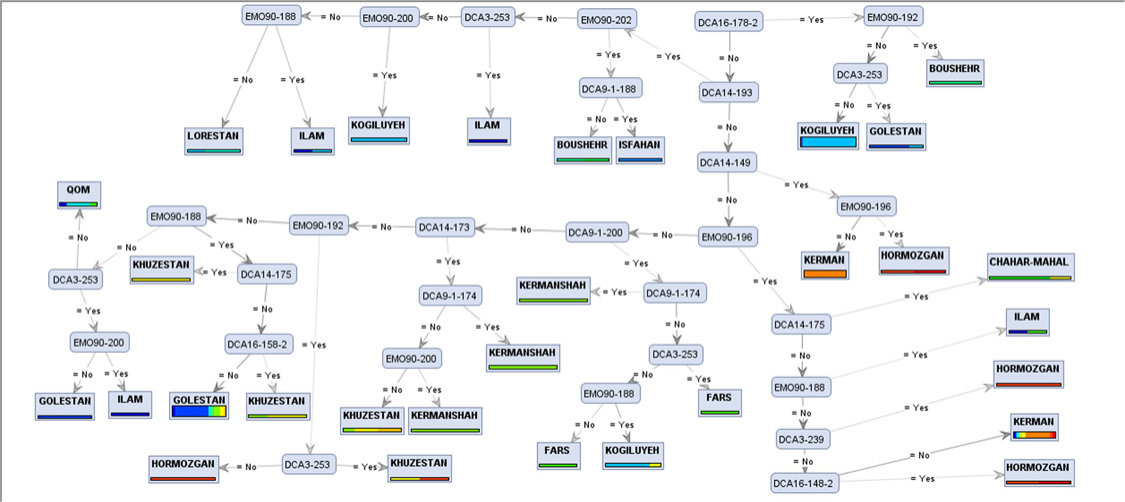
*Decision Tree* generated model showing separation of olive populations in the 16-targeted (16-t) experiment by different alleles. In this model, DCA-178 was selected as the main classifying attribute.

Tree induction is an efficient and popular method to construct classification models. These graphic models are easy to interpret and show which attributes are used to classify groups [11,13]. Decision trees are flexible and their branching complexity increases until the number of attributes (alleles) used to discriminate labels (populations) are sufficient [12,14]. In order to construct the most accurate decision trees, we applied four different tree induction algorithms of *Random Forest*, *Decision Tree*, *Decision Tree Parallel*, and *Decision Stump* [10,12] to the eight aforementioned datasets. Each algorithm ran with four different criteria of *Gain Ratio*, *Information Gain*, *Gini Index*, and *Accuracy* [9,41], using 10-fold cross validation. The Random Forest algorithm induces 10 different trees for each criterion, and the other algorithms each generate a single tree. Thus, 416 trees would be created in total for the eight datasets. In these generated trees (Figs 3 and 4), leaves (cornered rectangles) represent target (label) attributes and branches (rounded rectangles) represent attributes that lead to those labels.

The Naive Bayes classifier is a simple and effective inductive model of machine learning [9,15]. In order to achieve the best possible efficiency for machine-based prediction of olive populations [18,19], two algorithms of *Naive Bayes* (returns classification model using estimated normal distributions) and *Naive Bayes Kernel* (returns classification model using estimated Kernel densities) were trained with 10-fold cross validation [41] on all eight datasets.

A summary of the above procedure and the applied methods is presented in S1 Fig.

## Results

### Allele identification and allele frequency determination

For both 4-t and 16-t experiments, alleles across 11 microsatellite loci were screened. In the 4-t experiment with 267 accessions, DCA16 with 39 and EMO90 with 12 alleles were the most and the least variable loci, respectively (Tale 1). In total, 258 microsatellite alleles represented in 132 different tandem repeat lengths were observed. Allelic number (the average number of alleles per locus for all loci) ranged from 7.54 for reference cultivars to 17.54 for local ecotypes.

In the 16-t experiment with 169 accessions, DCA16 with 37 and EMO90 with 12 alleles were found to be the most and the least variable loci, respectively (Table 1). Totally 236 microsatellite alleles represented in 130 different tandem repeat lengths were observed. Allelic number ranged from 12.27 for *cuspidata* specimens to 17.54 for local ecotypes.

**Table 1:** Microsatellite allele lengths, loci and the total alleles.

Locus	Allele lengths (bp)	Total alleles
DCA3	227-**229**-232-235-237-239-241-243-245-247-249-251-253-255-257-**259**-261-263-**266**-270-272-274-277-279-281-283-286-288-**290**-293-295-**297**	32
DCA5	**192**-194-198-200-202-204-206-208-210-212-**214**-218-220-222-**228**-**234**	16
DCA9	162-164-166-169-170-172-174-176-178-180-182-184-186-188-190-192-194-196-198-200-202-204-206-208-210-214-216-218-220	29
DCA14	**143**-145-147-149-151-159-173-175-177-179-181-183-185-187-189-191-193-**197**	18
DCA16	122-124-126-128-130-**133**-135-137-139-142-144-146-148-150-152-154-156-158-160-162-**164**-166-170-172-174-176-178-180-182-184-**189**-**200**-**206**-**210**-**216**-218-220-**222**-**226**	39
DCA18	**159**-**161**-163-165-167-169-171-173-175-177-179-181-183-185-187-**191**-193-**195**-**197**-**198**-**207**	21
EMO-90	182-184-186-188-190-192-194-196-198-200-202-213	12
GAPU71B	118-121-122-124-126-127-128-130-132-134-136-138-140-142-144-146-**148**-**150**	18
GAPU101	182-**187**-191-193-195-197-199-201-203-**205**-207-209-215-217-219-221-**223**-**229**	18
GAPU103	134-136-139-141-144-146-148-150-154-157-159-**160**-162-**166**-168-172-174-177-179-181-184-186-188-190-192-**194**-**207**-**218**	28
UDO-043	**164**-168-**170**-172-174-176-178-180-**184**-186-188-190-**194**-**196**-198-**200**-202-204-206-208-210-212-214-216-218-220-222	27
Total		258

Alleles private to the Iranian accessions are highlighted in bold.

### Data cleaning

Among the 11 investigated loci, six loci with above 50% effective alleles were selected through the 4-t experiment as the most informative loci reserved for further analyses. These included DCA03, DCA09, DCA14, DCA16, UDO43 and EMO90. Throughout the data cleaning procedure, 157 alleles for the 4-t experiment and 149 alleles for the 16-t experiment were listed as effective alleles in FCdb (S2 Table).

### Selection of diagnostic alleles (feature selection)

The seven attribute weighting algorithms abovementioned were applied to the 4-t and 16-t datasets and produced results as follows:

### The 4-t experiment

Herein, totally 35 alleles out of 157 obtained weight values equal to or greater than 0.5 by at least one attribute weighting model. Among them, allele DCA14-149 was identified by all weighting algorithms as the most diagnostic allele. Alleles DCA16-150 and -154, EMO90-200 and **-**190, and DCA3-239 weighed high values by at least four of the seven models (S3 and S4 Tables).

### The 16-t experiment

Herein, totally 43 alleles out of 149 obtained weight values equal to or greater than 0.5 by at least one attribute weighting model. Among them, alleles DCA14-149, DCA16-178, and EMO90-188 were identified by all weighting algorithms as diagnostic (S3 and S4 Tables).

### Machine learning prediction of target populations

#### Tree induction models

For each experiment, 416 decision trees were generated by tree induction algorithms. Most of these algorithms were able to accurately distinguish among different labels (populations) with high efficiency. In the 4-t experiment, the highest accuracy (84.30%) was obtained when *Decision Tree* and *Decision Tree parallel* algorithms ran with *information gain* criterion on FCdb. Prediction rates for these algorithms are presented in Table 2, where 120 local ecotypes out of 132, 32 *cuspidata* specimens out of 37, 66 Mediterranean varieties out of 77, and 7 reference cultivars out of 21 were predicted correctly. However, 3 local ecotypes were predicted as *cuspidata* specimens, 5 of them as Mediterranean varieties, and 4 of them as reference cultivars. In addition, 12 reference cultivars were predicted as local ecotypes. It should be noted that similarity between reference cultivars and local ecotypes is expected due to their common origins.

**Table 2:** Prediction rate (accuracy) details of each decision tree with 10-fold cross validation for each of the populations in the 4-targeted (4-t) experiment, i.e. reference cultivars, Mediterranean varieties, cuspidata specimens, and local ecotypes.

	True	Local ecotypes	*cuspidata* specimens	Mediterranean varieties	Reference cultivars
Predicted					
**Local ecotypes**		120 (out of 132)	3	10	12
***cuspidata* specimens**		3	32 (out of 37)	1	1
**Mediterranean varieties**		5	2	66 (out of 77)	1
**Reference cultivars**		4	0	0	7 (out of 21)

Prediction rows indicate how records (olive accessions) were predicted by the model. True columns indicate how many records were predicted correctly.

In the 16-t experiment, the highest accuracy (61.7%) was obtained when *Decision Tree* and *Decision Tree parallel* algorithms ran with *gain ratio* criterion on *Chi Squared* dataset. Prediction rates for these algorithms are presented in Table 3, where 32 out of 36 samples from Kohgiluye & Boyerahmad, 23 out of 26 samples from Kerman, and 17 out of 23 samples from Golestan (these provinces have the highest number of samples among the 16 sampled provinces) were predicted correctly. Samples from Chaharmahal & Bakhtiari and Esfahan provinces were all predicted correctly. However, a high amount of admixture was revealed for the samples from other provinces.

**Table 3:** Prediction rate (accuracy) details of each decision tree with 10-fold cross validation for each of the types in the 16-targeted (16-t) experiment.

Sistan & Baluchestan	Hormozgan	Kerman	Zanjan	Khuzestan	Khorasan e Jonubi	Kermanshah	Fars	Charmahal & Bakhtiari	Yazd	Bushehr	Lorestan	Qom	Kohgiluye & Boyerahmad	Esfahan	Golestan	Ilam	True
																	Predicted
0	0	2	0	0	1	0	1	0	0	0	0	1	1	0	2	3	**Ilam**
0	0	0	0	2	0	3	1	0	0	0	2	0	0	0	17	4	**Golestan**
0	0	0	0	0	0	0	0	0	0	1	0	0	0	3	0	0	**Esfahan**
0	0	0	0	1	0	0	2	0	1	1	2	0	32	0	1	1	**Kohgiluye & Boyerahmad**
0	0	0	0	0	0	0	0	0	0	0	0	2	0	0	0	1	**Qom**
0	0	0	0	0	0	0	0	0	0	0	0	0	3	0	0	0	**Lorestan**
0	0	0	0	0	0	0	0	0	0	1	0	0	0	0	0	0	**Bushehr**
0	0	0	0	0	0	0	0	0	0	0	0	0	0	0	0	0	**Yazd**
0	0	0	0	1	0	0	0	3	0	0	0	0	0	0	0	0	**Charmahal & Bakhtiari**
0	0	0	0	0	0	2	2	0	0	0	0	0	0	0	0	0	**Fars**
0	0	0	1	2	0	7	0	0	0	0	0	0	0	0	1	0	**Kermanshah**
0	0	0	0	0	0	0	0	0	0	0	0	0	0	0	0	0	**Khorasan e Jonubi**
0	0	0	2	4	0	2	1	0	0	0	0	0	0	0	2	0	**Khuzestan**
0	0	0	0	1	0	0	0	0	0	0	0	0	0	0	0	0	**Zanjan**
3	4	23	0	1	0	0	1	0	0	0	0	1	0	0	0	3	**Kerman**
0	6	0	0	1	0	1	0	0	0	1	0	0	0	0	0	0	**Hormozgan**
0	0	1	0	0	0	0	0	0	0	0	0	0	0	0	0	0	**Sistan & Baluchestan**

Prediction rows indicate how records (olive accessions) were predicted by the model. True columns indicate how many records were predicted correctly.

Performances of all algorithms are presented in S5 Table. It is visible in this table that the selected alleles were diagnostic enough to characterize different olive accessions from different provinces. For each dataset, the model with the best performance is presented in Figs 3 and 4.

For the 4-t experiment, most of the graphic models showed a high level of complexity. This was due to high similarity between labels, so that more branches had to be generated to distinguish among olive populations based on many attributes. The induced tree selected allele EMO90-200 as the main attribute, and used it together with allele DCA14-149 to categorize *cuspidata* specimens.

Fig 3 illustrates the tree constructed by the *Decision Tree* model based on FCdb. DCA14-149 was selected as the root of the tree, by which any accession would be categorized in the *cuspidata* specimen population. Meanwhile, presence of any of the three alleles DCA14-149, -147, and -145 would help to separate *cuspidata* specimens from other populations. Accessions that do not show any of these alleles but represent any of the alleles DCA16-154, DCA3-239, EMO90-190, or UDO43-208 would be categorized as Mediterranean varieties. The two other populations were too complicated for assigning attributes to them.

Fig 4 represents the induced decision tree by *Decision Tree* model with *Information Gain* criterion on FCdb for the 16-t experiment. Allele DCA16-178 was used as a diagnostic attribute to build root for the constructed decision trees. In combination with allele EMO90-192, the tree was able to identify cultivars from Bushehr province, while absence of allele DCA16-178 combined with presence of alleles DCA14-193, EMO90-202 and DCA9-188 identified accessions from Esfahan province.

### Naive Bayes models

The best performance obtained by *Naive Bayes* models on the eight datasets of the 4-t experiment was 90.98%, which was achieved when *Naive Bayes* and *Naive Bayes Kernel* ran on FCdb (S6 Table). Table 4 shows more details of the population prediction, confirming that 115 out of 132 local ecotypes, 75 out of 77 Mediterranean varieties, and 16 out of 21 reference cultivars were correctly identified. Besides, all 37 samples of *cuspidata* specimens were correctly identified.

**Table 4:** Prediction rate (accuracy) details of each Bayesian algorithm with 10-fold cross validation for each of the populations in the 4-targeted (4-t) experiment, i.e. reference cultivars, Mediterranean varieties, cuspidata specimens, and local ecotypes.

	True	Local ecotypes	*cuspidata* specimens	Mediterranean varieties	Reference cultivars
Predicted					
**Local ecotypes**		115 (out of 132)	0	2	5
***cuspidata* specimens**		0	37 (out of 37)	0	0
**Mediterranean varieties**		5	0	75 (out of 77)	0
**Reference cultivars**		12	0	0	16 (out of 21)

Prediction rows indicate how records (olive accessions) were predicted by the model. True columns indicate how many records were predicted correctly.

In the 16-t experiment, (S6 Table), the performance is still remarkable bearing in mind that due to a probable history of interbreeding, close genetic relationships among different groups of olive accessions belonging to one provincial area is expected. However, the applied model almost precisely detected even the slight allelic differences among these accessions.

As presented in Table 5, by using *Naive Bayes* algorithms, olive accessions from Esfahan, Bushehr, and Charmahal & Bakhtiari were identified with 100% accuracy, while accessions from Sistan & Baluchestan, Khorasan e Jonubi, and Yazd remained unclassified.

**Table 5:** Prediction rate (accuracy) details of each Bayesian algorithm with 10-fold cross validation for each of the populations in the 16-targeted (16-t) experiment.

Sistan & Baluchestan	Hormozgan	Kerman	Zanjan	Khuzestan	Khorasan e Jonubi	Kermanshah	Fars	Charmahal & Bakhtiari	Yazd	Bushehr	Lorestan	Qom	Kohgiluye & Boyerahmad	Esfahan	Golestan	Ilam	True
																	Predicted
0	0	0	0	0	0	0	1	0	0	0	0	0	1	0	3	8	**Ilam**
0	0	0	0	0	0	0	0	0	0	0	2	2	0	0	16	2	**Golestan**
0	0	0	0	0	0	0	0	0	0	0	0	0	0	3	0	0	**Esfahan**
0	0	0	0	0	0	1	0	0	0	0	0	0	35	0	1	1	**Kohgiluye & Boyerahmad**
0	0	0	0	0	0	0	0	0	0	0	0	2	0	0	0	0	**Qom**
0	0	0	0	0	0	0	0	0	0	0	2	0	0	0	1	0	**Lorestan**
0	0	0	0	0	0	0	0	0	1	4	0	0	0	0	0	0	**Bushehr**
0	0	0	0	0	0	0	0	0	0	0	0	0	0	0	0	0	**Yazd**
0	0	0	0	1	0	0	0	3	0	0	0	0	0	0	0	0	**Charmahal & Bakhtiari**
0	0	0	0	1	0	0	6	0	0	0	0	0	0	0	1	0	**Fars**
0	0	0	1	1	0	10	0	0	0	0	0	0	0	0	1	0	**Kermanshah**
0	0	1	0	0	0	0	0	0	0	0	0	0	0	0	0	0	**Khorasan e Jonubi**
0	0	1	1	10	0	3	1	0	0	0	0	0	0	0	0	1	**Khuzestan**
0	0	0	1	0	0	1	0	0	0	0	0	0	0	0	0	0	**Zanjan**
2	3	21	0	0	1	0	0	0	0	0	0	0	0	0	0	0	**Kerman**
1	6	2	0	0	0	0	0	0	0	0	0	0	0	0	0	0	**Hormozgan**
0	1	1	0	0	0	0	0	0	0	0	0	0	0	0	0	0	**Sistan & Baluchestan**

Prediction rows indicate how records (olive accessions) were predicted by the model. True columns indicate how many records were predicted correctly.

## Discussion

We are reporting the most informative microsatellite loci which may contribute to the classification of the Iranian and the Mediterranean olive gene pool. Six loci (DCA03, DCA09, DCA14, DCA16, UDO43, and EMO90) from a starting set of eleven loci were selected based on their efficiency in characterizing the four populations in this study. The informative features of UDO43 and DCA16 have been reported in previous studies [30,33,42–44]. Alba *et al*. showed that UDO43 and DCA16 loci are able to differentiate 30 olive cultivars from southern Italy, without ascertaining synonyms among them [45]. Baldoni *et al*. also reported that UDO43 is the most indicative locus among 77 Mediterranean cultivars [33]. Several studies have employed these markers for identification and characterization of genomic regions in olives [25,30,34,35,46,47]. However, finding ranked patterns/combinations of markers which may provide higher efficiencies for differentiating among olive populations has not been attempted before. Supervised pattern recognition models, in particular decision tree models, are methods of choice for this purpose, which can outperform the common multivariate methods. This is the first study, to the best of our knowledge, which is reporting use of supervised machine learning methods and predictive models to find the best indicative combination of candidate microsatellite markers. Our results distinguished olive accessions from geographically separate regions with high accuracy and performance via introducing the most effectively differentiating alleles among different populations.

When the number of target groups was raised from the first (4-t) to the second (16-t) experiment, an increase in the number of informative loci was observed. The diagnostic alleles reported previously [30,33,42–44] were also supported by the machine learning models used in this study. Our data can serve as an identification assay for discriminating olive populations based on specific diagnostic alleles. Concerning the statistical basis of the decision tree models, markers such as DCA14-149, DCA9-206, and DCA16-178-2 which are located at the top of the tree hierarchies (Figs 3 and 4) are the key discriminating markers which shape the topology, and construct patterns of the marker-based discrimination.

According to Hosseini-Mazinani *et al*., analyses showed that microsatellite alleles are shared among *cuspidata* specimens and local ecotypes and/or reference cultivars [21]. They also documented that a few local ecotypes from Fars and Charmahal & Bakhtiari possess alleles that are able to characterize all *cuspidata* specimens. This study revealed that 50.27% of the alleles are shared between Iranian reference cultivars/local ecotypes and Mediterranean varieties, while only 24.73% of alleles are shared in direct comparison of Mediterranean varieties and *cuspidata* specimens and 7.19% in a comparison of reference cultivars/local ecotypes with *cuspidata* specimens. Herein, we introduce a method of dissection by which accessions at the presence of any of the three alleles DCA14-149, -147 and -145 are classified as *cuspidata* specimens, representing separate populations. This offers a highly useful diagnostic tool for further studies of *cuspidata* populations.

Bayesian algorithms were even more successful than the decision trees in predicting and categorizing accessions within the four expected populations, as *Naive Bayes* and *Naive Bayes Kernel* retrieved an accuracy of 90.98% (S6 Table). The details of the prediction rate for each population showed that the reference cultivar is a complex group with high similarity to other populations. The other three populations had a few false predictions and could be separated with high accuracy. For example, within the local ecotype population 115 out of 132 accessions could be correctly assigned, which gives an accuracy of 87% (Table 2). The explained details show that although the provincial accessions are highly analogous, they can be conveniently predicted by combined microsatellite results and the machine learning models (Table 4).

Hosseini-Mazinani *et al*. reported a clear separation between Mediterranean and Iranian olive samples based on Bayesian analysis of the population structure. However, the latter group represented by both local ecotypes and reference cultivars was shown to be completely admixed [21]. While previous studies gave an overall image of polymorphism across the studied populations, the present study provided details on this diversity by assessing the effectiveness of the polymorphic loci in the characterization of those populations by employing useful machine learning methods. Naive Bayes algorithms showed a better performance than the tree induction models in the 16-t experiment, i.e. the experiment involving different provinces. Taking into account the high number of labels (16), Naive Bayes produced the relatively high performance accuracy of 75.26%. This highlights the power of Naive Bayes in differentiating olive populations. The prediction details are presented in Table 5.

Tree induction in the second experiment (Fig 4) was very complex, providing 61.70% accuracy which is a performance higher than expected. Remarkably, Bushehr, Qom, Lorestan, and Charmahal & Bakhtiari were the provinces from which all accessions could be identified through one pathway (i.e. all their accessions were classified in one group). These pathways had branches varying in number from two branches for Bushehr to nine branches for Qom. However, other provinces required more than one pathway (varying from two to four pathways), which means their accessions are diverse and classified in different groups.

## Conclusion

Our data indicate that application of pattern discovery models on microsatellite markers is a novel tool for phylogeographic characterization of different populations of olive. In this study, through a combination of finely-selected microsatellite markers and analytic and predictive methods we developed a rapid, precise and cost-effective characterizing assay for olive accessions. In addition, candidate alleles were assessed based on their merits in discriminating different geographical groups of accessions. Using both attribute weighting and machine learning models and based on pattern discovery, olive accessions from separate regions were accurately classified by only microsatellite marker information. Given that no geographical attribute was defined in our analyses, this classification revealed specific gene pools in each province presented by more than 90% accuracy using Naive Bayes algorithm, as well as exchanges of genetic material among other provinces which are not so distinctly isolated. Accurate identification of accessions from several provinces is an indication for the presence of distinct populations throughout the Iranian plateau. Considering an old olive growing tradition within this region and taking into account the complex propagation pathways of olive as an ancient crop species, more paleontology and molecular studies, including association studies using the methodology developed in this study, are suggested to shed further light on current structure and historical dynamics of olive gene pools.

## Supporting Information

S1 Fig Methodology flowchart. showing methods and algorithms applied to the investigation of microsatellite (SSR) markers in this study.
(PDF)

S1 Table Names of the loci/primer pairs and their range of allele lengths given in an order of informative merit; adapted from Baldoni *et al*. [33]. (PDF)

S2 Table List of all 157 selected alleles which remained after data cleaning. (PDF)

S3 Table List of diagnostic alleles obtaining a weight value equal to or greater than 0.5 by at least four weighting algorithms in the 4-targeted (4-t) and 16-targeted (16-t) experiments. (PDF)

S4 Table Complete attribute weighting results for the 4-targeted (4-t) and 16-targeted (16-t) experiments. (XLS)

S5 Table Complete decision tree results for the 4-targeted (4-t) and 16-targeted (16-t) experiments. (XLS)

S6 Table Calculated prediction rate (accuracy) of each Bayesian algorithm in 4-targeted (4-t) and 16-targeted (16-t) experiments, shown separately for each of the eight datasets, i.e. each Attribute Weighted dataset (AWds) and Final Cleaned database (FCdb). (PDF)
