# Supplementary figures and images for: Machine Learning Based Classification of Microsatellite Variation: An Effective Approach for Phylogeographic Characterization of Olive Populations
Source: PLoS One. 2015 Nov 24;10(11):e0143465. doi: 10.1371/journal.pone.0143465 (PMC4658005; doi:10.1371/journal.pone.0143465)

**S1 Fig.**

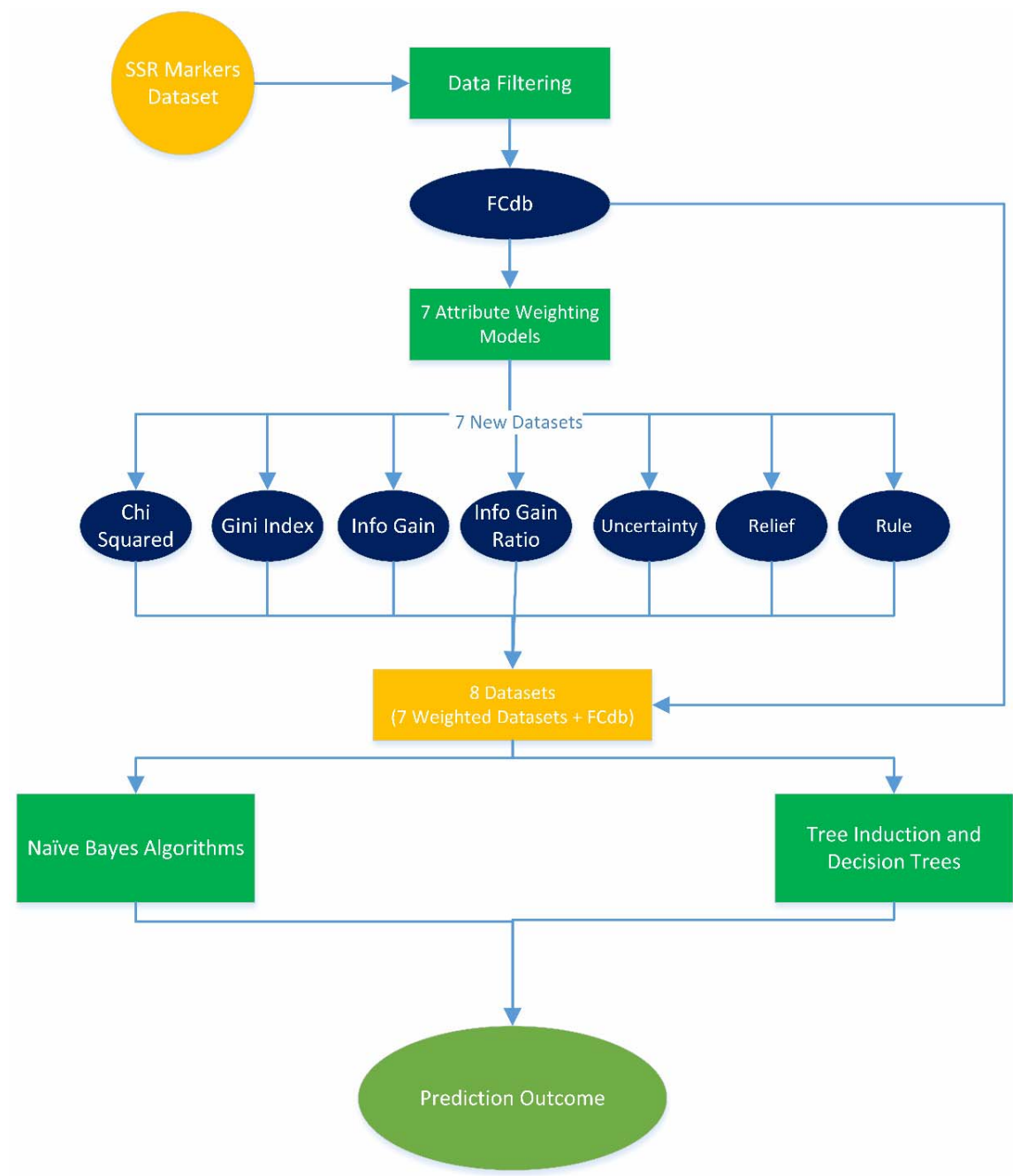

Supplement: S1 Fig — showing methods and algorithms applied to the investigation of microsatellite (SSR) markers in this study. (PDF) [file pone.0143465.s001.pdf]
