# Supplementary material for: Machine Learning Based Classification of Microsatellite Variation: An Effective Approach for Phylogeographic Characterization of Olive Populations
Source: PLoS One. 2015 Nov 24;10(11):e0143465. doi: 10.1371/journal.pone.0143465 (PMC4658005; doi:10.1371/journal.pone.0143465)
Supplement: S1 Table — (PDF) [file pone.0143465.s002.pdf]

S1 Table.

Locus/primer pair	Minimum–maximum allele lengths (bps)	Merit order
UDO-43	172–216	1
DCA-09	162–210	2
GAPU-103A	136–190	3
DCA-18	163–187	4
DCA-16	124–174	5
GAPU-101	182–218	6
DCA-03	232–255	7
GAPU-71B	121–144	8
DCA-05	194–208	9
DCA-14	173–191	10
EMO-90	186–198	11
