# Supplementary material for: Machine Learning Based Classification of Microsatellite Variation: An Effective Approach for Phylogeographic Characterization of Olive Populations
Source: PLoS One. 2015 Nov 24;10(11):e0143465. doi: 10.1371/journal.pone.0143465 (PMC4658005; doi:10.1371/journal.pone.0143465)
Supplement: S2 Table — (PDF) [file pone.0143465.s003.pdf]

S2 Table.

DCA3-227	DCA14-189	DCA16-124	UDO43-184
DCA3-229	DCA14-191	DCA16-126	UDO43-186
DCA3-232	DCA14-193	DCA16-128	UDO43-188
DCA3-235	DCA14-197	DCA16-130	UDO43-190
DCA3-237	EMO90-182	DCA16-133	UDO43-194
DCA3-239	EMO90-184	DCA16-135	UDO43-196
DCA3-241	EMO90-186	DCA16-137	UDO43-198
DCA3-243	EMO90-188	DCA16-139	UDO43-200
DCA3-245	EMO90-190	DCA16-142	UDO43-202
DCA3-247	EMO90-192	DCA16-144	UDO43-204
DCA3-249	EMO90-194	DCA16-146	UDO43-206
DCA3-251	EMO90-196	DCA16-148	UDO43-208
DCA3-253	EMO90-198	DCA16-150	UDO43-210
DCA3-255	EMO90-200	DCA16-152	UDO43-212
DCA3-257	EMO90-202	DCA16-154	UDO43-214
DCA3-259	EMO90-213	DCA16-156	UDO43-216
DCA3-261	DCA9-162	DCA16-158	UDO43-218
DCA3-263	DCA9-164	DCA16-160	UDO43-220
DCA3-266	DCA9-166	DCA16-162	
DCA3-270	DCA9-169	DCA16-164	
DCA3-272	DCA9-170	DCA16-166	
DCA3-274	DCA9-172	DCA16-170	
DCA3-277	DCA9-174	DCA16-172	
DCA3-279	DCA9-176	DCA16-174	
DCA3-281	DCA9-178	DCA16-176	
DCA3-283	DCA9-180	DCA16-178	
DCA3-286	DCA9-182	DCA16-180	
DCA3-288	DCA9-184	DCA16-182	
DCA3-290	DCA9-186	DCA16-184	
DCA3-293	DCA9-188	DCA16-189	
DCA3-295	DCA9-190	DCA16-200	
DCA3-297	DCA9-192	DCA16-206	
DCA14-143	DCA9-194	DCA16-210	
DCA14-145	DCA9-196	DCA16-216	
DCA14-147	DCA9-198	DCA16-218	
DCA14-149	DCA9-200	DCA16-220	
DCA14-151	DCA9-202	DCA16-222	
DCA14-159	DCA9-204	DCA16-226	
DCA14-173	DCA9-206	UDO43-164	

DCA14-175	DCA9-208	UDO43-168	
DCA14-177	DCA9-210	UDO43-170	
DCA14-179	DCA9-214	UDO43-172	
DCA14-181	DCA9-216	UDO43-174	
DCA14-183	DCA9-218	UDO43-176	
DCA14-185	DCA9-220	UDO43-178	
DCA14-187	DCA16-122	UDO43-180	
