# Supplementary material for: Machine Learning Based Classification of Microsatellite Variation: An Effective Approach for Phylogeographic Characterization of Olive Populations
Source: PLoS One. 2015 Nov 24;10(11):e0143465. doi: 10.1371/journal.pone.0143465 (PMC4658005; doi:10.1371/journal.pone.0143465)
Supplement: S3 Table — (PDF) [file pone.0143465.s004.pdf]

S3 Table.

16-t experiment		4-t experiment	
**No. of selective attribute weightings (out of 10)**	**Attribute**	**No. of selective attribute weightings (out of 10)**	**Attribute**
7	DCA14-149	7	DCA14-149
7	DCA16-178	5	DCA16-154
7	EMO90-188	5	EMO90-200
6	DCA9-1-200	4	DCA16-150
5	EMO90-200	4	DCA3-239
5	DCA14-193	4	EMO90-190
5	DCA3-253		
4	EMO90-196		
4	UDO43-176		
4	UDO43-214		
