# Supplementary material for: Machine Learning Based Classification of Microsatellite Variation: An Effective Approach for Phylogeographic Characterization of Olive Populations
Source: PLoS One. 2015 Nov 24;10(11):e0143465. doi: 10.1371/journal.pone.0143465 (PMC4658005; doi:10.1371/journal.pone.0143465)
Supplement: S6 Table — (PDF) [file pone.0143465.s007.pdf]

S6 Table.

16-t experiment		4-t experiment		
Naive Bayes Kernel	***Naive Bayes***	***Naive Bayes Kernel***	***Naive Bayes***	**Dataset**
67.57%	67.57%	60.70%	60.70%	**Chi Squared**
75.26%	75.26%	90.98%	90.98%	**FCdb**
53.27%	53.27%	81.28%	81.28%	**Gini Index**
62.90%	62.90%	78.32%	78.32%	**Info Gain**
63.93%	63.93%	70.80%	70.80%	**Info Gain Ratio**
61.65%	61.65%	70.40%	70.40%	**Relief**
58.75%	58.75%	80.17%	80.17%	**Rule**
65.85%	65.85%	79.42%	79.42%	**Uncertainty**
